# Biophysical Characteristics Reveal Neural Stem Cell Differentiation Potential

**DOI:** 10.1371/journal.pone.0025458

**Published:** 2011-09-30

**Authors:** Fatima H. Labeed, Jente Lu, Hayley J. Mulhall, Steve A. Marchenko, Kai F. Hoettges, Laura C. Estrada, Abraham P. Lee, Michael P. Hughes, Lisa A. Flanagan

**Affiliations:** 1 Centre for Biomedical Engineering, University of Surrey, Guildford, United Kingdom; 2 Department of Neurology and Sue and Bill Gross Stem Cell Research Center, University of California Irvine, Irvine, California, United States of America; 3 Department of Biomedical Engineering, University of California Irvine, Irvine, California, United States of America; 4 Department of Pathology and Laboratory Medicine, University of California Irvine, Irvine, California, United States of America; 5 Laboratory for Fluorescence Dynamics, University of California Irvine, Irvine, California, United States of America; University of São Paulo, Brazil

## Abstract

**Background:**

Distinguishing human neural stem/progenitor cell (huNSPC) populations that will predominantly generate neurons from those that produce glia is currently hampered by a lack of sufficient cell type-specific surface markers predictive of fate potential. This limits investigation of lineage-biased progenitors and their potential use as therapeutic agents. A live-cell biophysical and label-free measure of fate potential would solve this problem by obviating the need for specific cell surface markers.

**Methodology/Principal Findings:**

We used dielectrophoresis (DEP) to analyze the biophysical, specifically electrophysiological, properties of cortical human and mouse NSPCs that vary in differentiation potential. Our data demonstrate that the electrophysiological property membrane capacitance inversely correlates with the neurogenic potential of NSPCs. Furthermore, as huNSPCs are continually passaged they decrease neuron generation and increase membrane capacitance, confirming that this parameter dynamically predicts and negatively correlates with neurogenic potential. In contrast, differences in membrane conductance between NSPCs do not consistently correlate with the ability of the cells to generate neurons. DEP crossover frequency, which is a quantitative measure of cell behavior in DEP, directly correlates with neuron generation of NSPCs, indicating a potential mechanism to separate stem cells biased to particular differentiated cell fates.

**Conclusions/Significance:**

We show here that whole cell membrane capacitance, but not membrane conductance, reflects and predicts the neurogenic potential of human and mouse NSPCs. Stem cell biophysical characteristics therefore provide a completely novel and quantitative measure of stem cell fate potential and a label-free means to identify neuron- or glial-biased progenitors.

## Introduction

Stem cells self-renew and generate progenitors capable of further differentiation into specialized cell types. Neural stem cells of the cerebral cortex generate glia (astrocytes and myelinating oligodendrocytes) and an array of phenotypically distinct neurons. Developmental neurobiology studies of the rodent cortex identified resident stem cells that predominantly generate neurons at early stages of brain formation and glia at later stages, suggesting the presence of neuron-biased (or potentially neuron-restricted) progenitors at early stages and progenitors linked to glial fates at later times [1], [2], [3], [4], [5], [6]. Human brain development has been more difficult to study, but data suggest that cortical formation in humans is more complex than that in rodents since the human cortex is not simply a larger, expanded version of the rodent cortex [7]. The pattern of early neuron generation and later gliogenesis appears to also hold true for human cortical development, but neuron-biased progenitors may be present in the human cortex from very early stages of embryonic development (5–6 weeks gestation) and persist through mid-gestational stages (14–23 weeks gestation) [8], [9], [10]. Neuronal progenitors populate an outer subventricular zone not present in rodents and may have greater proliferative potential than intermediate progenitors in the rodent subventricular zone [11]. Despite species differences, evidence exists for neuronal and glial progenitors in both rodents and humans.

HuNSPC cultures, whether arising from cells isolated from cortical tissue or from differentiation of human embryonic stem cells, are heterogeneous populations of stem and more committed progenitor cells and contain both neuronal and glial progenitors. Specific cues such as growth factors [12], [13], glutamate [14], [15], and extracellular matrix molecules [16] enhance neurogenesis or in some cases encourage generation of a particular type of neuron from cultured huNSPCs. These cues may increase neuronal, rather than glial, progenitors by specifically affecting the fate decisions of cells in the population or by stimulating the selective expansion of neuronal progenitors. Despite clear in vivo and in vitro evidence for the existence of neuron-biased progenitors, little is known about their cellular characteristics and the factors that distinguish them from their glial-biased counterparts. Identification of human cortical progenitors biased to particular differentiated cell fates would enable further insight into the critical processes underlying neuro- and gliogenesis during human brain formation.

Progenitors linked to specific fates are of interest as cell therapeutics for human central nervous system injuries and diseases since transplantation of a biased progenitor would increase the likelihood that a specific population of differentiated cells is formed. The production of differentiated neurons or oligodendrocytes from transplanted cells would replenish these specialized cell types that are often lost to illness or injury, but unlike astrocytes are not efficiently replaced by endogenous sources of cells. Formation of neurons or oligodendrocytes may be preferred in some transplant situations, but NSPCs not biased to a specific differentiated cell fate will predominantly produce astrocytes after transplantation into the damaged or diseased brain [17], [18], [19], a response that is likely due to cues manufactured by damaged tissue. A different and potentially life-threatening problem is presented by populations of undifferentiated cells that resist differentiation after transplant and can eventually form tumors [20]. One way to ensure controlled differentiation of transplanted cells is to utilize progenitors biased to the fate of interest that are less plastic and are fully committed to differentiation.

Phenotypically distinct subtypes of living cells are usually identified by methods such as flow cytometry or fluorescence activated cell sorting (FACS) that rely on the availability of surface antigens unique to the cells of interest. Despite clear evidence for neuron-biased progenitors from developmental studies, there has been difficulty in using marker-based methods for their isolation. Available cell surface biomarkers such as PSA-NCAM, A2B5, CD133, LeX, and CD24 do not sufficiently distinguish neuronal and glial progenitors due to a lack of specificity that includes recognition of more or less differentiated cells in the lineage [21], [22], [23], [24], [25]. An approach for identifying and isolating cells that does not rely on specific cell surface markers would aid efforts to characterize fate-biased human progenitor cells.

Biophysical characteristics of living cells, such as electrophysiological parameters, can be determined without markers and thus provide an alternative and complementary means to identify cell phenotype. Whole cell electrophysiological properties contribute to the behavior of cells in dielectrophoresis (DEP) [26], which induces motion of particles or cells in non-uniform AC electric fields in a frequency-dependent manner. Characterization of cells by DEP has successfully enabled detection of cancerous and apoptotic human cells before they express indicative cell surface markers [27], [28], [29], [30], [31]. Electrophysiological properties measured by DEP may reveal neural cell phenotype since we previously found that the behavior of differentiated mouse neurons and astrocytes in DEP differ from each other and from undifferentiated mouse NSPCs. Furthermore, mouse neuron- and glial-biased progenitors are distinguished by DEP and adopt dielectric signatures similar to those of the differentiated progeny they will become (e.g. the response of astrogenic NSPCs to DEP is more similar to that of astrocytes than neurons) [32]. At lower DEP frequencies, astrogenic NSPCs experience positive DEP, which is defined by the movement of cells toward electrodes in response to an inhomogeneous electric field, while neurogenic NSPCs experience negative DEP and are repelled from electrodes. The behavior of mouse NSPCs in DEP suggests that whole cell biophysical measures could prove useful for determining progenitor lineage commitment and specific electrophysiological characteristics that distinguish neuron- and astrocyte-biased progenitors might exist [32].

The crossover frequency of cells provides a quantitative measure of their frequency-dependent responses to the electric fields in DEP. Furthermore, cells with distinct crossover frequencies can be separated using DEP. A cell's crossover frequency is defined as the frequency at which the cell has no net movement in relation to the DEP electrodes because there is no net induced DEP force (i.e. the cell does not experience positive or negative DEP). Cells that have different crossover frequencies can be separated by choosing frequencies that produce positive DEP in one cell type and negative DEP in the other [33], [34], [35], [36], [37]. The cells experiencing positive DEP are attracted toward electrodes while those in negative DEP will be repelled from electrodes, thus creating a differential force for separation.

Since a biophysical measure of NSPC cell fate potential would aid understanding of neuro- and gliogenesis as well as the use of fate-specific huNSPCs for therapeutic purposes, we analyzed human and mouse NSPC electrophysiological characteristics using DEP. We tested NSPCs that were similar in size and morphology but differ in ability to form neurons and astrocytes. Our analyses focused on membrane electrophysiological parameters because our previous results showed that the responses of mouse NSPCs to DEP frequencies ranging from 10–1000 kHz distinguished cells of different fate potential [32] and tenets of dielectrophoretic theory suggest that cellular responses in this frequency range are dominated by the plasma membrane [35]. We also analyzed NSPC crossover frequencies since cells that differ in this parameter can be separated by DEP. Our data show the electrophysiological property membrane capacitance, but not membrane conductance, is a specific and dynamic indicator of NSPC fate potential. Furthermore, NSPCs that differ in fate potential have distinct crossover frequencies, suggesting that DEP may be used to isolate undifferentiated NSPCs based on their propensity to form either neurons or glia.

## Results

**Figure 1:**
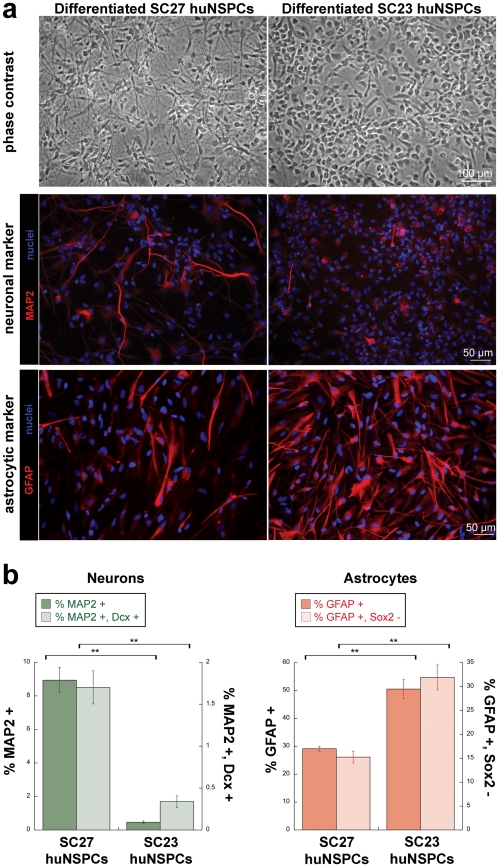
HuNSPCs differ in neurogenic and gliogenic potential. (a) Differentiated SC27 (left panels) and SC23 (right panels) huNSPCs are shown by phase contrast microscopy (top panels) and immunostaining for the neuronal marker MAP2 (middle panels, all cell nuclei stained blue) or the astrocytic marker GFAP (bottom panels, all cell nuclei stained blue). Images are not from the same field. (b) Percentages of differentiated neurons and astrocytes generated from SC27 and SC23 huNSPCs were quantified. Neuron counts were obtained from cells differentiated for 14 days and labelled for the neuronal markers MAP2 and doublecortin (Dcx). Cells with processes positive for MAP2 or doubly-positive for MAP2 and Dcx are expressed as a percentage of the total cells (**p<0.01, n = 5000 or more cells from 3 or more independent experiments). Astrocytes were quantified after differentiation for 7 days. Cells positive for GFAP or GFAP-positive and sox2-negative are expressed as a percentage of the total cells (**p<0.01, n = 1000 or more cells from 3 or more independent experiments). Error bars represent s.e.m.

We tested the differentiation potential of two unique sets of huNSPCs isolated at equivalent stages of gestation [16], [38] to determine whether they might differ in propensity to form differentiated neurons and glia and could be used to test the hypothesis that stem cell electrophysiological properties reflect biases in fate potential. In order to quantitatively determine progenitor cell fate potential, we induced differentiation of SC27 and SC23 huNSPCs and characterized differentiated cell phenotypes as an indicator of the fate potential of the progenitors. We measured generation of neurons and astrocytes, but not oligodendrocytes since they are not efficiently generated by either set of cells in our usual differentiation conditions [39]. In comparison to SC23 huNSPCs, SC27 cells generated numerous cells with compact cell bodies and extensive processes that resembled neurons by phase contrast microscopy (Fig. 1a, top panels) and expressed the neuronal marker MAP2 (Fig. 1a, middle panels). In contrast, SC27 cells formed fewer cells that stained with the astrocytic marker GFAP than SC23 cells (Fig. 1a, bottom panels).

The identities of cells differentiated from huNSPCs were confirmed by determining the presence of neuron and astrocyte markers by immunostaining and assessing cell morphological criteria. We counted the percentages of cells that extended long processes and expressed the neuronal marker MAP2 or, for a more stringent standard of neuronal phenotype, co-expressed two neuronal markers (MAP2 and doublecortin)(Materials and Methods and Fig. S1a). In both cases quantitation revealed that SC27 huNSPCs generate greater numbers of neurons than SC23 cells (Fig. 1b). Undifferentiated cortical stem/progenitor cells can express the typical astrocytic marker GFAP (e.g. [40]), making it difficult to distinguish these cells from astrocytes. We labelled cells with GFAP or a combination of GFAP and sox2, a stem/progenitor cell marker, to determine the numbers of GFAP-positive cells (Fig. 1b), true astrocytes (GFAP-positive and sox2-negative)(Fig. 1b and Fig. S1b), putative progenitor cells (GFAP-positive and sox2-positive)(Fig. S1b), and stem/progenitor cells (GFAP-negative and sox2-positive) (Fig. S1b). SC27 huNSPCs had a lower propensity to generate astrocytes (Fig. 1b) and GFAP-positive/sox2-positive cells (Fig. S1b) than SC23 cells, but had higher levels of cells that were only sox2-positive than SC23 cells (Fig. S1b). Quantitation of differentiated cell phenotypes revealed statistically significant differences in the neuron and astrocyte differentiation potential of SC27 and SC23 huNSPCs (Fig. 1b). SC27 and SC23 cells therefore could be used to test the hypothesis that electrophysiological properties indicate fate potential of undifferentiated huNSPCs.

**Figure 2:**
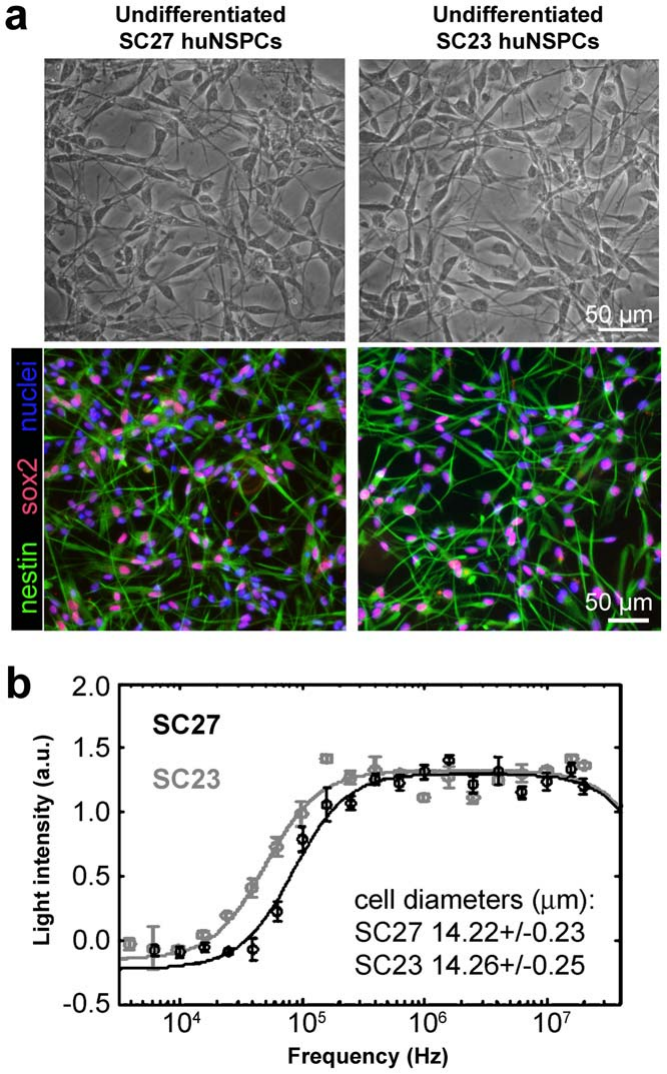
Unique sets of undifferentiated huNSPCs that are similar in morphology and marker analysis differ in dielectric properties. (a) Undifferentiated SC27 and SC23 huNSPCs are grown as adherent cultures on fibronectin (top panels, phase contrast images) and similarly express the stem cell markers nestin and sox2 (bottom panels, fluorescent images). Phase contrast and fluorescent images are not of the same field. (b) Representative DEP spectra of SC27 (black) and SC23 (gray) cells are shown over a range of frequencies. The circles on the graph represent measured data, whilst the line represents the DEP spectrum derived from the best-fit model (n = 5 measurements from the same set of cells in a single experiment, error bars represent s.d.). Light intensity reflects cells' positions in the well after application of the DEP force and is mathematically related to the dielectric properties of the cells (see Materials and Methods) [41]. SC27 and SC23 huNSPCs do not differ in average cell diameter (inset, p = 0.90, n = 170 or more cells from 3 or more independent experiments, values are means +/− s.e.m.).

Although SC27 and SC23 huNSPCs vary in differentiation potential, the undifferentiated cells are remarkably similar. Both sets of cells were isolated at the same stage of gestation, have comparable morphology (Fig. 2a, top panels), and express similar levels of the stem cell markers nestin and sox2 ([16] and Fig. 2a, bottom panels). The undifferentiated NSPCs were in suspension when assessed by DEP to determine electrophysiological properties and there was no significant difference in the diameters of SC27 and SC23 cells in suspension (SC27 14.22+/−0.23 µm, SC23 14.26+/−0.25 µm, p = 0.90). Further analysis using confocal microscopy determined that there was no difference in cell perimeters of SC27 and SC23 cells (SC27 55.0+/−2.6 µm, SC23 52.1+/−1.8 µm, p = 0.37). The similarities in diameter and perimeter suggest that SC27 and SC23 cells do not significantly differ in morphology when in suspension as used for DEP measurements. Both sets of huNSPCs are viable over several hours in the low ionic strength iso-osmotic buffer used for DEP (Fig. S1c), as previously demonstrated for mouse NSPCs and encompassing the time necessary for DEP analysis of electrophysiological properties [32].

Electrophysiological measurements of undifferentiated NSPCs were obtained using the DEP-Well system as described in detail previously [41] (see also Materials and Methods). Briefly, the responses of NSPCs to DEP forces were assessed over a full spectrum of frequencies ranging from 2 kHz to 20 MHz and DEP spectra acquired for each set of cells. DEP spectra of SC27 and SC23 huNSPCs demonstrate unique responses of the cells to specific DEP frequencies (Fig. 2b), which is not due to size differences between the cells since their diameters are almost identical (SC27 vs. SC23, p = 0.90; inset, Fig. 2b). Our previous work indicated that neurogenic mouse NSPCs experience positive DEP and are attracted to electrodes at higher frequencies than gliogenic mouse NSPCs, resulting in DEP curves for neurogenic cells that are right-shifted compared to those for their gliogenic counterparts [32]. The huNSPCs analyzed here demonstrated the same pattern; SC27 cells that are more neurogenic produced DEP spectra that are right-shifted compared to the spectra of SC23 cells.

**Figure 3:**
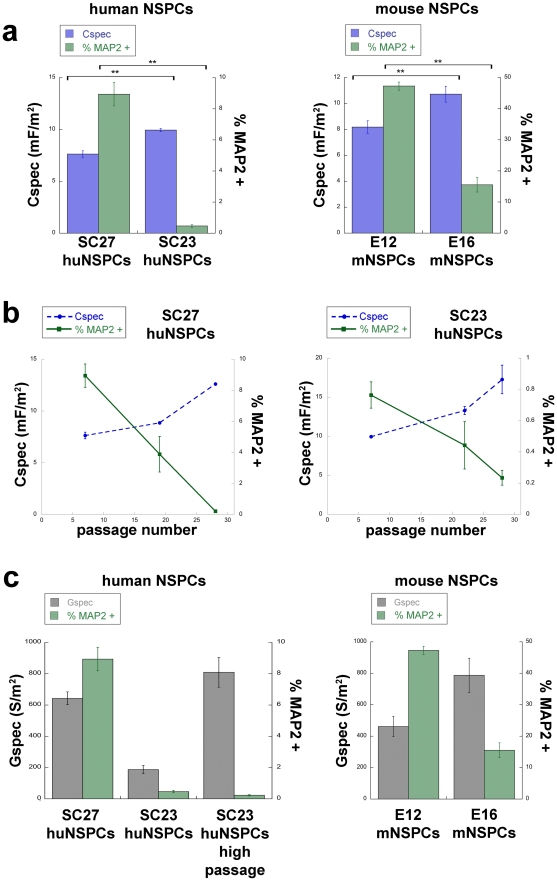
NSPC membrane capacitance correlates with neurogenic potential but membrane conductance does not. (a) Graphs show that the specific membrane capacitance (Cspec, mF = milliFarad) of undifferentiated NSPCs is inversely correlated with the ability of the cells to generate neurons (quantified as the percentage of MAP2-positive cells with extended processes after differentiation). Left panels are data from SC27 and SC23 huNSPCs and right panels are E12 and E16 mouse NSPCs (mNSPCs) (**p<0.01, n = 3 or more separate experiments with different sets of cells). (b) Membrane capacitance (Cspec) values of undifferentiated SC27 (left panel) and SC23 (right panel) cells increase over passage number (dashed blue line), concomitant with a decrease in the generation of neurons from these cells (solid green line). Significance values are reported in the text. (c) Specific membrane conductance (Gspec, S = Siemens) of undifferentiated human and mouse NSPCs does not consistently correlate with their neurogenic potential as shown by the generation of MAP2-positive cells with extended processes after differentiation (n = 3 or more separate experiments with different sets of cells). High passage SC23 huNSPCs are passage 25–28. Error bars in all graphs represent s.e.m.

Measured DEP spectra of huNSPCs were fit to the single shell dielectric model [42] to determine specific membrane capacitance (Cspec) and conductance (Gspec) values for the cells [43]. These calculations take cell size into account, so the values of capacitance and conductance are cell size independent. Membrane capacitance is a measure of the ability of the membrane to store charge and generate a dipole in a frequency-dependent manner in DEP and is governed by both morphology and composition. The membrane capacitance values of SC27 and SC23 huNSPCs were significantly different from each other, with values for SC27 cells ∼23% lower than those of SC23 cells (SC27 7.6+/−0.3 mF/m^2^ vs. SC23 9.9+/−0.2 mF/m^2^, p<0.01). These cells are similar in many regards but differ in fate potential, suggesting that the membrane capacitance of huNSPCs inversely correlates with their neurogenic capacity (Fig. 3a). Neurogenic fate potential in Figure 3 is shown by MAP2 staining of differentiated cells since this single phenotypic marker reflects the difference in fate potential between SC27 and SC23 huNSPCs shown by multiple neuronal markers (Fig. 1b) and multiple marker analysis of the cells analyzed in Figure 3 gave the same results (data not shown).

To further test the association of membrane capacitance with NSPC neurogenic capacity, we analyzed mouse NSPCs that differ in ability to generate neurons. Mouse NSPCs isolated from the cerebral cortex at earlier stages of embryonic development (embryonic day 12, E12) formed more neurons upon differentiation than cells isolated at a later developmental stage (embryonic day 16, E16)(Fig. S2). E12 and E16 mouse NSPCs were of similar size (E12 11.6+/−0.6 µm; E16 12.0+/−0.3 µm, p = 0.56, n = 20 or more cells) and show no differences in the expression of nestin, sox2, or GFAP [32]. Neurogenic mouse NSPCs exhibited lower membrane capacitance than their gliogenic counterparts (E12 8.2+/−0.5 mF/m^2^ vs. E16 10.7+/−0.6 mF/m^2^), showing that membrane capacitance also reflects fate bias of mouse NSPCs (Fig. 3a). The inverse correlation between membrane capacitance and neurogenic potential of both human and mouse NSPCs shows that membrane capacitance is a label-free electrophysiological parameter that indicates NSPC fate bias.

The neurogenic capacity of huNSPCs decreases with continued passaging, so we used cells at different passage numbers to test whether huNSPC membrane capacitance dynamically predicts changes in cell fate potential. With increasing passage number, SC27 and SC23 huNSPCs significantly decrease neuron generation and increase membrane capacitance (Fig. 3b, Cspec SC23 p7 vs. p22 p<0.01, p7 vs. p28 p<0.01, n = 3 or more separate experiments with different sets of cells; %MAP2-positive SC23 p7 vs. p28 p<0.05; SC27 p7 vs. p19 p<0.01, p7 vs. p28 p<0.01, p19 vs. p28 p<0.01, n = 6000 or more cells from 3 or more separate experiments). These data from two different sets of cells confirm that the biophysical parameter membrane capacitance continues to inversely correlate with and successfully predict huNSPC neurogenic potential as it dynamically shifts over continued cell passaging.

Although membrane capacitance correlates with neurogenic potential of NSPCs, membrane conductance does not. Membrane conductivity describes the potential of the membrane to transmit charge. When analyzed across several human and mouse NSPCs that differ in fate potential, the differences in specific membrane conductivity per unit area (Gspec) did not consistently correlate with the ability of the cells to generate neurons (Fig. 3c). Furthermore, analysis of Gspec and neurogenic potential over passage of huNSPCs failed to show a consistent correlation between these measures (Fig. S3). These findings suggest that the electrophysiological property membrane capacitance, rather than membrane conductance, reflects the fate potential of both human and mouse NSPCs.

**Figure 4:**
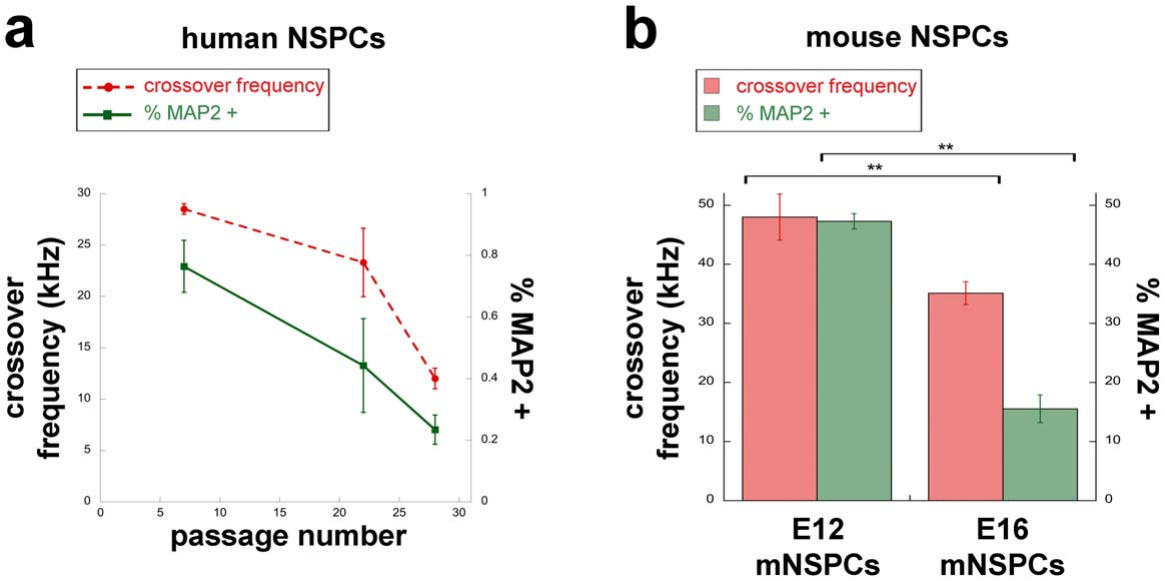
NSPC crossover frequency reflects neurogenic potential. (a) The crossover frequency of undifferentiated SC23 huNSPCs decreases with passage number (dashed red line) and directly correlates with neurogenic potential of the cells as shown by their ability to generate differentiated process-bearing cells expressing MAP2 (solid green line). Significance values are reported in the text. (b) The crossover frequency of undifferentiated mouse E12 and E16 NSPCs directly correlates with the ability of the cells to generate neurons (shown as the percentage of MAP2-positive cells with extended processes after differentiation). Error bars in all graphs represent s.e.m., **p<0.01, n = 3 or more separate experiments with different sets of cells.

The DEP spectra of neurogenic human NSPCs (Fig. 2b) and DEP trapping curves of neurogenic mouse NSPCs [32] occur at higher frequencies than those of their respective gliogenic counterparts, making it possible that NSPCs with different fate biases have unique crossover frequencies (frequency at which there is no net induced DEP force). The crossover frequency is of particular interest since DEP can separate cells that differ in this parameter. We used the changing fate potential of huNSPCs over passaging to test whether the crossover frequency can reflect huNSPC fate bias. The crossover frequency of huNSPCs significantly changed over increasing passage number and directly correlated with neuron generation from the cells (Fig. 4, crossover frequency huNSPC p7 vs. p28 p<0.01, p22 vs. p28 p<0.05, n = 3 or more separate experiments with different sets of cells; the cells in Fig. 4a are the same as shown in Fig. 3b so the %MAP2-positive cells are included again here to demonstrate the correlation of crossover frequency with neurogenic ability). The size of the cells did not significantly change over passage number (SC23 diameter at p7 was 14.7+/−0.4 µm and at p28 was 15.7+/−0.4 µm, p = 0.094; similarly, SC27 diameter at p7 was 14.6+/−0.3 µm and at p28 was 14.7+/−0.4 µm, p = 0.823;) and computer simulations predicted that a difference in diameter of more than 10 µm would be necessary to shift the crossover frequency as seen for the SC23 cells at different passages (J. Lu, unpublished data). We tested whether the positive association of crossover frequency with neurogenic potential also applies to mouse NSPCs and found that neurogenic E12 mouse NSPCs had a higher crossover frequency than E16 gliogenic mouse NSPCs (Fig. 4b). These data show that the direct correlation of crossover frequency with neuronal fate bias holds true for both human and mouse NSPCs.

## Discussion

Inherent cell properties that do not require the use of specific labels for detection provide a novel means to identify progenitors biased to particular differentiated cell fates. The data presented here identify membrane capacitance as a specific electrophysiological property that reflects the fate bias of human and mouse NSPCs. Membrane capacitance correlates inversely with neurogenic potential and directly with astrogenic potential of NSPCs. The association of the electrophysiological properties of both human and mouse NSPCs with fate potential shows that this is not a species-specific phenomenon. Additionally, human NSPC membrane capacitance shifts with changing fate potential of the cells, indicating that it is a dynamic measure of cell fate. Membrane capacitance is a novel, quantitative and label-free indicator of NSPC phenotype and fate potential that can be employed to identify fate-specific NSPCs for research and therapeutic purposes.

The human and mouse NSPC membrane capacitance values measured in our study are within the range of those reported for rat NSPCs using patch clamp techniques [44], [45], [46], although there are inherent differences in how membrane capacitance is measured by DEP and patch clamp. Whole-cell measurements of capacitance as obtained by DEP are dependent on the surface area and thickness of the plasma membrane (hence the amount of total membrane present) whereas patch clamp capacitance values reflect only the membrane patch under observation [31], [47]. Furthermore, DEP-measured capacitance reflects changing cell status since cells undergoing apoptosis or terminal differentiation exhibit shifts in membrane capacitance [29], [47], while capacitance measured by patch clamp is considered more constant [48]. The membrane capacitance values measured by DEP for huNSPCs are within the lower range of those measured for rat NSPCs using patch clamp. Conversion of our capacitance numbers determined by DEP to the same units reported by patch clamp (pF, picoFarad) yields values ranging from 5–13 pF for human NSPCs, which are within the range of 5–23 pF reported in the literature for rat NSPCs (23 pF for E10.5 rat NSPCs [44], 9 pF mean value with range 5–15 pF for E15 rat NSPCs [46], and 13 pF for E16 rat hippocampal NSPCs [45]). Although the two techniques differ, the values for NSPC membrane capacitance we report using DEP are not dissimilar to those obtained by patch clamp.

Distinct membrane capacitance measurements for neuron- and astrocyte-biased progenitors might point to specific plasma membrane characteristics associated with these cell types. A variety of extracellular factors influence the generation of neurons from huNSPCs [12], [13], [15], [16] and the cell surface receptors for these ligands may contribute to the distinct membrane capacitance of neurogenic huNSPCs. However, previous studies suggest that at the DEP frequencies used here (10 to 1000 kHz), membrane capacitance may not be sensitive to physiological expression of a transmembrane protein [48], [49] or resting membrane potential [50]. In contrast, membrane capacitance would be affected by the surface area of the membrane and sensitive to membrane topology. Molecular structures extending from the membrane surface [51] and lipid composition, including macromolecular structures such as lipid rafts, would affect the cell membrane structure. Interestingly, significant changes in the glycosylation of membrane components that would alter cell membrane structure occur between neurogenic and gliogenic time points in cortical development [52], [53]. Future studies will be necessary to determine whether these shifts in glycosylation patterns are the biological basis for the distinct electrophysiological signatures of fate-biased progenitors. Links between membrane capacitance and fate potential of NSPCs may lead to identification of specific membrane components that define neurogenic and gliogenic progenitors.

Our data show that the DEP spectra of neurogenic huNSPCs occur at higher frequencies than the spectra of gliogenic cells (Fig. 2). We previously reported the same pattern for mouse cells; the DEP trapping curves of neuron-biased mouse NSPCs are at higher frequencies that the curves of gliogenic mouse NSPCs [32]. These patterns indicate that neurogenic cells reach the crossover frequency, which is the frequency at which cells shift from negative to positive DEP and a quantitative measure of the frequency-dependent response of cells in DEP, at higher frequencies than gliogenic NSPCs. Support for the association of crossover frequency with NSPC fate potential is provided by data demonstrating dynamic changes in crossover frequency that reflect the changing fate potential of huNSPCs over passaging (Fig. 4). Furthermore, mouse NSPCs that differ in fate potential also significantly differ in crossover frequency (Fig. 4). The higher frequency dielectric signatures of both human and mouse neurogenic NSPCs suggest that fate-biased progenitors begin to adopt characteristics of the cells they will eventually form upon differentiation since the DEP curves of neurons are at higher frequencies (50% cells in positive DEP at 500 kHz) while those of astrocytes are at lower frequencies (50% cells in positive DEP at 75 kHz) [32], [36]. NSPC dielectric properties and crossover frequency thus provide a label free and quantitative measure of fate potential that reflects cellular changes occurring as cells become tied to a particular differentiated fate but prior to detectable expression of markers associated with the differentiated cell phenotypes.

The association of crossover frequency with fate potential of NSPCs suggests that DEP may be useful for separating NSPCs that are biased to a particular differentiated cell lineage. Cells that have different crossover frequencies can be separated from each other by DEP by choosing frequencies that produce positive DEP in one cell type and negative DEP in the other cell type. The cells experiencing positive DEP are attracted toward electrodes while those in negative DEP will be repelled from electrodes, thus creating a differential force for separation. Cells separated and purified by DEP include neurons from astrocytes, subpopulations of human leukocytes, and stimulated vs. quiescent Jurkat cells [33], [34], [35], [36], [37]. In our ongoing studies we are exploring DEP as a label-free separation method for neural lineage progenitors.

DEP is generally non-toxic [54], [55], but for it to be useful as a separation tool for NSPCs it is important that the DEP buffer and electric fields do not alter NSPC survival, proliferation, or differentiation. Incubation in DEP buffer for several hours does not alter human (Fig. S1) or mouse [32] NSPCs. We find that DEP electric field exposure for the short time frame necessary for separation, or for the time needed to collect the data presented here, does not alter survival, proliferation, or differentiation for either human or mouse NSPCs (data not shown). Since toxicity is not an issue, future isolation of fate-biased NSPCs by DEP will make it possible to analyze the cellular characteristics that distinguish neurogenic from gliogenic progenitors. Furthermore, DEP-isolated NSPCs that differ in fate potential can be easily assessed in models of CNS injury and disease since the cells are not labelled by antibodies for separation and therefore can be immediately transplanted. Transplantation experiments will be critical for assessing the regenerative capacity of unique populations of huNSPCs isolated by DEP.

We report unique membrane electrophysiological characteristics of human neuron-biased progenitors that distinguish them from their astrocyte-biased counterparts without the use of markers. A whole-cell quantitative biophysical indicator of stem cell differentiation potential preceding detectable changes in cell biochemical makeup is a novel concept likely to apply to many stem cell populations. Additionally, cell membrane capacitance provides a classification of stem and more differentiated cells that is complementary to more traditional tools used to examine these cells and is objective and noninvasive. Stem cell biophysical properties indicative of phenotype may lead to the discovery of global cell membrane changes with specific functional consequences that are associated with particular cell phenotypes. Human stem cell membrane biophysical properties predictive of cell differentiation potential as described here have the potential to significantly advance scientific insights into neural stem cell differentiation and the use of specific progenitor cells in therapeutic applications.

## Materials and Methods

### Ethics statement

Informed written consent was obtained for all human subjects and all human cell research has been approved by the University of California, Irvine Institutional Review Board and involves cells with no patient identifiers. All research involving mouse cells has been approved by the University of California, Irvine Institutional Animal Care and Use Committee (2001–2304) and University of California, Irvine has an approved Animal Welfare Assurance (A3416-01) on file with the NIH Office of Laboratory Animal Welfare.

### Cell culture and differentiation

#### Human NSPCs

Two fetal-derived human neural stem/progenitor cell (huNSPC) cultures (SC23 and SC27) were isolated from the cerebral cortices of two separate brains at the same gestational age (23 weeks) and maintained as previously described [16], [38]. Briefly, undifferentiated cells were grown as adherent cultures on fibronectin coated flasks in basal medium [DMEM/F12 (Invitrogen, Carlsbad, CA), 20% (v/v) BIT-9500 (Stem Cell Technologies, Vancouver BC), 1% (v/v) antibiotic/antimycotic (Invitrogen, Carlsbad, CA)] supplemented with the following mitogenic growth factors: 40 ng/ml EGF (BD Biosciences, Bedford, MA), 40 ng/ml FGF (BD Biosciences, Bedford, MA), and 40 ng/ml PDGF (Peprotech, Rocky Hill, NJ). HuNSPCs were passaged approximately every 7 days using Cell Dissociation Buffer (Invitrogen, Carlsbad, CA) and split 1∶2. Cells were isolated for most experiments at low passage (p7–10), but at mid (p19–22) or high passage (p25–28) for select experiments.

For neuronal differentiation, huNSPCs were plated on laminin-coated coverslips [16] in 1∶1 basal medium and Neurobasal medium (Invitrogen, Carlsbad, CA) supplemented with 1× B27 (Invitrogen, Carlsbad, CA), 1% heat inactivated fetal bovine serum (Invitrogen, Carlsbad, CA), 20 ng/ml BDNF (Peprotech, Rocky Hill, NJ), 20 ng/ml NT3 (Peprotech, Rocky Hill, NJ), 2.5 ng/ml FGF (BD Biosciences, Bedford, MA), and 0.1 µM retinoic acid (Sigma, St. Louis, MO) for at least 14 days. To induce astrocyte differentiation, huNSPCs on laminin-coated coverslips were incubated in DMEM/F12 (Invitrogen, Carlsbad, CA), 20% (v/v) heat-inactivated FBS (Invitrogen, Carlsbad, CA) and 1% (v/v) antibiotic/antimycotic (Invitrogen, Carlsbad, CA) for at least 7 days.

#### Mouse NSPCs

Culture conditions for mouse fetal-derived cortical NSPCs (mNSPCs) have been reported previously [16], [32]. In short, mNSPCs were cultured from brain cortical regions of wild type CD1 mice at embryonic days 12.5 and 16.5 (referred to as E12 and E16). Cells were grown as neurospheres in DMEM, 1× B27, 1× N2, 1 mM sodium pyruvate, 2 mM glutamine, 1 mM N-acetyl-cysteine (Sigma, St. Louis, MO), 20 ng/ml EGF (BD Biosciences, Bedford, MA), 10 ng/ml FGF (BD Biosciences, Bedford, MA), and 2 µg/ml heparin (Sigma, St. Louis, MO). For differentiation, mNSPCs were plated on laminin-coated coverslips in media lacking growth factors and heparin for 3 days.

### Cell preparation prior to DEP experiments

A single cell suspension of huNSPCs was prepared for DEP analysis using Cell Dissociation Buffer (Invitrogen, Carlsbad, CA). Cells were washed twice with and resuspended into DEP buffer, an iso-osmotic medium consisting of 8.5% (w/v) sucrose (Sigma, St. Louis, MO), 0.3% (w/v) glucose (Sigma, St. Louis, MO) [56], and adjusted to a final conductivity of 100 µS/cm using 150 mM KCl (Sigma, St. Louis, MO). Buffer conductivity was measured with a conductivity meter (RS components Ltd, London, UK). Cell counts and viability for each experiment were determined using trypan blue exclusion. Viability of human NSPCs in the DEP buffer was assessed by trypan blue exclusion after incubating the cells in suspension at room temperature for up to 4 hours. Viable cells were expressed as a percentage of the total cells. For DEP measurements the final cell concentration was adjusted to approximately 3×10^6^ cells/mL. In order to reduce the effect of variation in cell number in each sample, the experiments were repeated at least four times with different populations.

For confocal microscopy, cell membranes of huNSPCs (SC23 and SC27) were labelled with 2 µM DiI (Molecular Probes, C7001) in Hank's balanced salt solution (HBSS) for 5 min at 37°C then for 15 min at 4°C. After labeling, the cells were returned to regular media. Fluorescently labeled cells in solution (in DEP buffer as prepared for DEP experiments) were examined with laser scanning confocal microscopy (Olympus FV1000) using a 543 nm laser at ∼300 nm resolution. Images of cells were analyzed in ImageJ to measure cell perimeters (n = 20 or more cells per measurement).

Mouse NSPC neurospheres were dissociated using NeuroCult dissociation buffer (Stem Cell Technologies, Vancouver BC) to form a single cell suspension for DEP analysis. Washing and resuspension of cells in DEP buffer prior to DEP experiments was as described for human NSPCs.

### DEP measurements

DEP measurements were obtained using the DEP-Well system, as described in detail by Hoettges et al. [41]. Cells were placed in wells with dimensions similar to those in a 1536-well plate, but bearing 12 ring-shaped, 17 µm-wide, gold-plated copper electrodes around the well circumference with gaps of 75 µm between electrodes. Electrodes were energized with AC (alternating 10V_pk-pk_ or ground along the electrode sequence) at specific frequencies. The well was placed on a microscope stage and light from the microscope passing through the well documents the position of cells within the well as cells are either attracted to the electrodes along the walls of the well (positive DEP) or repelled from the walls and collect at the center (negative DEP). The magnitude of the corresponding change in light absorbance across the well due to the local concentration of cells in the column is mathematically related to the polarisability of the cell population. The radial nature of the system means that there is a direct correlation between change in light intensity from a uniformly dispersed case as the field is applied and the value of polarisability of the cells in the well.

The well was observed using a Nikon inverted microscope equipped with a 1.3 Mpixel video camera, and the change in light intensity across the well over time was determined using a MATLAB (The Mathworks Inc, Natick, MA) script. The change in cell distribution was monitored by recording an image every 3 s for a total of 60 s and 21 images. The well was energized with frequencies ranging from 1 kHz–20 MHz at 5 points per decade. We focused our analysis on these frequencies to determine the dielectric properties of the membrane compartment of the cells since cytoplasmic contributions to the DEP response were at frequencies higher than 20 MHz (the outer limit of our function generator). Using MATLAB, light intensity measurements were fit to the single shell model (as in [43]) and the best-fit model (minimum line correlation coefficient 0.98) was used to determine the specific membrane capacitance (Cspec), specific membrane conductance (Gspec), and crossover frequency (the frequency where the DEP force is zero). For DEP electrophysiology calculations, cell diameters were measured using either ImageJ software to analyze microscope images of cells in a hemocytometer or the Countess automated cell counter (Invitrogen, Carlsbad, CA).

### Immunofluorescence staining and quantitation

To determine the differentiation potential of the stem cell populations, NSPCs were induced to differentiate and the generation of neurons and astrocytes assessed. Immunostaining was as previously described [16] and used the following antibodies: anti-doublecortin (C-18) polyclonal, 1∶100 (Santa Cruz Biotechnology, Santa Cruz, CA); anti-GFAP (clone G5A) monoclonal, 1∶200 (Sigma, St. Louis, MO); anti-MAP2 (microtubule-associated protein 2) (clone HM2) monoclonal, 1∶100 (Sigma, St. Louis, MO); anti-class III beta-tubulin (TuJ1) polyclonal, 1∶5000 (Research Diagnostics, Flanders, NJ), the TuJ1 antibody was used for mouse cells only as there was significant non-specific staining of the human cells with this antibody; anti-sox2 (Y-17) polyclonal, 1∶100 (Santa Cruz Biotechnology, Santa Cruz, CA). The secondary antibodies were donkey anti-mouse Alexa-555, donkey anti-goat Alexa-488, and donkey anti-rabbit Alexa-488, all 1∶100 (Molecular Probes/Invitrogen, Carlsbad, CA). Percentages of cells that differentiated into neurons or astrocytes were calculated from images of randomly selected fields for each cell population and used nuclei stained by Hoechst 33342 (2 µg/ml in phosphate-buffered saline, Molecular Probes, Eugene, OR) to determine the total cell number. Controls included cells stained with secondary antibodies only (negative controls) and appropriate subcellular localization of antibody signal (for example, nuclear for sox2 transcription factor and cytoskeletal for GFAP intermediate filament protein).

Stringent criteria were applied to assess generation of neurons and astrocytes from huNSPCs since the morphologies of the differentiated cells are not always as distinctive as they are for mouse cells. Furthermore, many of the commercially available antibodies for human neuronal markers show cross-reactivity with non-neuronal cell types. For neuronal assessments we only counted cells that extended processes at least 3× the length of the cell body and either expressed the neuronal marker MAP2 or co-expressed MAP2 and an additional neuronal marker, doublecortin. As shown in Figure 1, fewer cells are co-stained with both neuronal markers but the differences in neurogenic capacity between SC27 and SC23 huNSPCs are similar whether shown by MAP2 expression alone or MAP2/doublecortin co-expression. Astrocytes were distinguished from stem/progenitor cells that express GFAP by only counting cells that were GFAP-positive and negative for sox2, which is expressed by stem/progenitor cells. All statistical analyses used two-tailed unpaired Student's t-tests with normal data distribution. Significance values and sample sizes are reported for each analysis in the text or figure legends.

## Supporting Information

Figure S1 **HuNSPCs differentiate into neurons that are double-stained with neuronal markers, differ in astrogenic capacity, and are viable in the DEP buffer.** (a) SC27 huNSPCs differentiated for 14 days and immunostained for MAP2 and doublecortin reveal process-bearing neurons that double-stain for both markers. Doublecortin staining tends to be strongest at the tips of neuronal processes. Similar neurons were observed in differentiated SC23 cells, although in fewer numbers. (b) SC27 and SC23 huNSPCs differ in the generation of astrocytes (GFAP-positive but sox2-negative cells, red bars, **p<0.01, n = 1300 or more cells), putative progenitors (GFAP-positive and sox2-positive cells, blue bars, *p<0.05, n = 1300 or more cells), and undifferentiated stem/progenitor cells (sox2-positive but GFAP-negative cells, gray bars, **p<0.01, n = 1300 or more cells) after differentiation for 7 days. (c) Dissociated SC23 and SC27 huNSPCs incubated in DEP buffer (see Supporting Information Methods) exhibit no significant loss in viability over 4 hours, which is considerably longer than the time necessary for the completion of DEP experiments with the cells. Cell viability was assessed by trypan blue staining and live cells expressed as a percentage of the total cells. Error bars represent s.e.m.
(TIF)

Figure S2 **Mouse NSPCs (mNSPCs) from distinct stages of development (E12 and E16) differ in neurogenic capacity.** (a) Immunostaining of differentiated mNSPCs from distinct developmental stages with antibodies to neuronal markers reveals that more MAP2-positive neurons are generated from E12 mNSPCs than E16 cells. Similar results were obtained with antibodies for the neuronal markers TuJ1 and doublecortin (data not shown). (b) The percentages of differentiated MAP2-positive neurons and GFAP-positive astrocytes generated from E12 and E16 mNSPCs shows that E12 cells are more neurogenic and E16 cells more gliogenic. Neuron and astrocyte counts were obtained from cells differentiated for 3 days (**p<0.01, n = 250 or more cells from 3 separate experiments). Error bars represent s.e.m.
(TIF)

Figure S3 **HuNSPC membrane capacitance does not consistently correlate with neurogenic potential.** Graphs of specific membrane conductance (Gspec, S = Siemens) and neurogenic capacity of SC27 or SC23 cells (revealed by generation of MAP2-positive neurons) over increasing passage number demonstrate the lack of a clear correlation between these measures. Gspec values of SC27 cells begin high, drop lower, and increase again as the neurogenic potential decreases. SC23 cells display a similar decrease in neurogenic potential over increasing passage number but their Gspec values begin low, increase slightly, and increase again. Error bars represent s.e.m. and n = 3 or more separate experiments with different sets of cells.
(TIF)
