# Supplementary figures and images for: Biophysical Characteristics Reveal Neural Stem Cell Differentiation Potential
Source: PLoS One. 2011 Sep 30;6(9):e25458. doi: 10.1371/journal.pone.0025458 (PMC3184132; doi:10.1371/journal.pone.0025458)

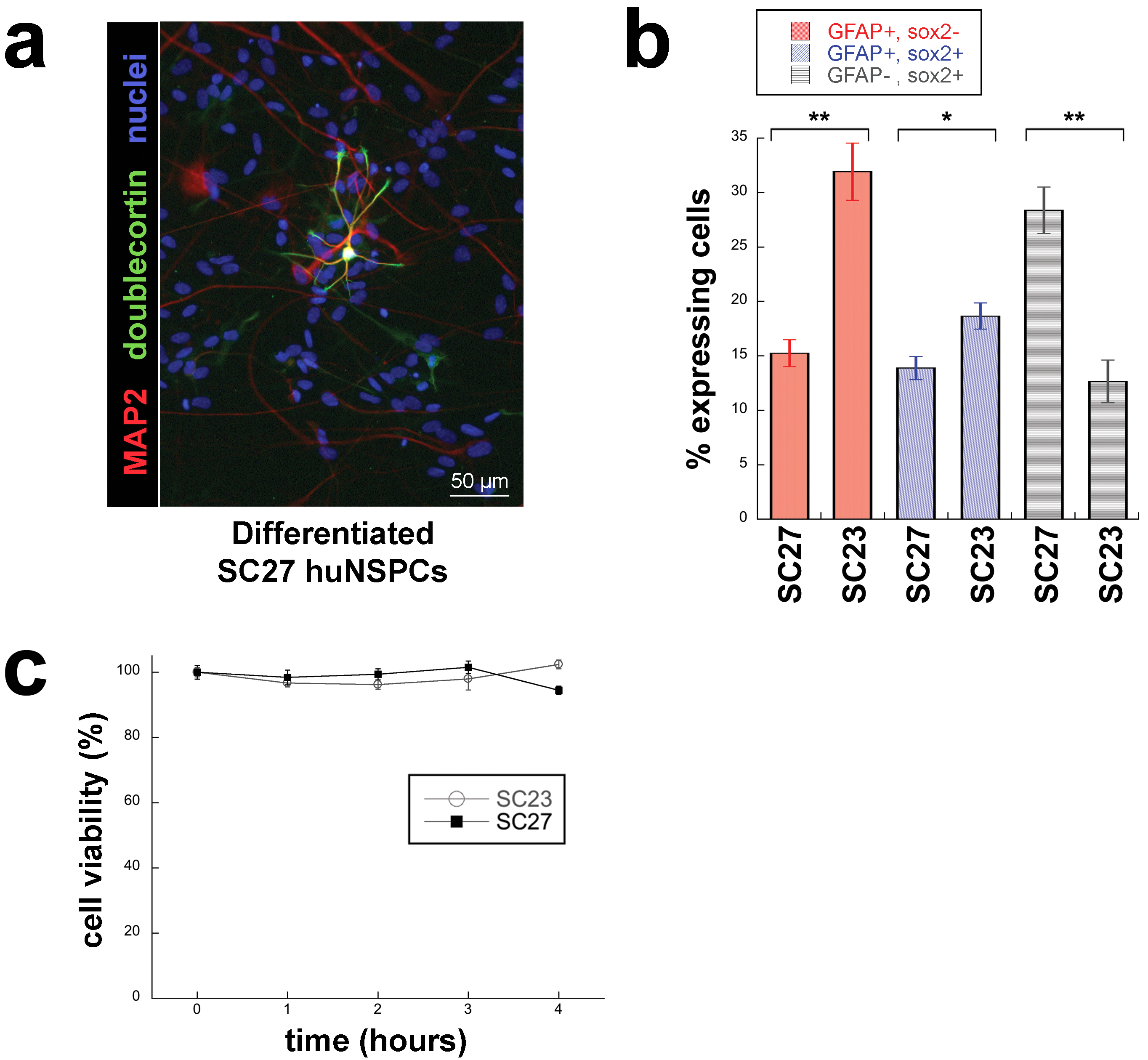

Supplement: Figure S1 — HuNSPCs differentiate into neurons that are double-stained with neuronal markers, differ in astrogenic capacity, and are viable in the DEP buffer. (a) SC27 huNSPCs differentiated for 14 days and immunostained for MAP2 and doublecortin reveal process-bearing neurons that double-stain for both markers. Doublecortin staining tends to be strongest at the tips of neuronal processes. Similar neurons were observed in differentiated SC23 cells, although in fewer numbers. (b) SC27 and SC23 huNSPCs differ in the generation of astrocytes (GFAP-positive but sox2-negative cells, red bars, **p<0.01, n = 1300 or more cells), putative progenitors (GFAP-positive and sox2-positive cells, blue bars, *p<0.05, n = 1300 or more cells), and undifferentiated stem/progenitor cells (sox2-positive but GFAP-negative cells, gray bars, **p<0.01, n = 1300 or more cells) after differentiation for 7 days. (c) Dissociated SC23 and SC27 huNSPCs incubated in DEP buffer (see Supporting Information Methods) exhibit no significant loss in viability over 4 hours, which is considerably longer than the time necessary for the completion of DEP experiments with the cells. Cell viability was assessed by trypan blue staining and live cells expressed as a percentage of the total cells. Error bars represent s.e.m. (TIF) [file pone.0025458.s001.tif]

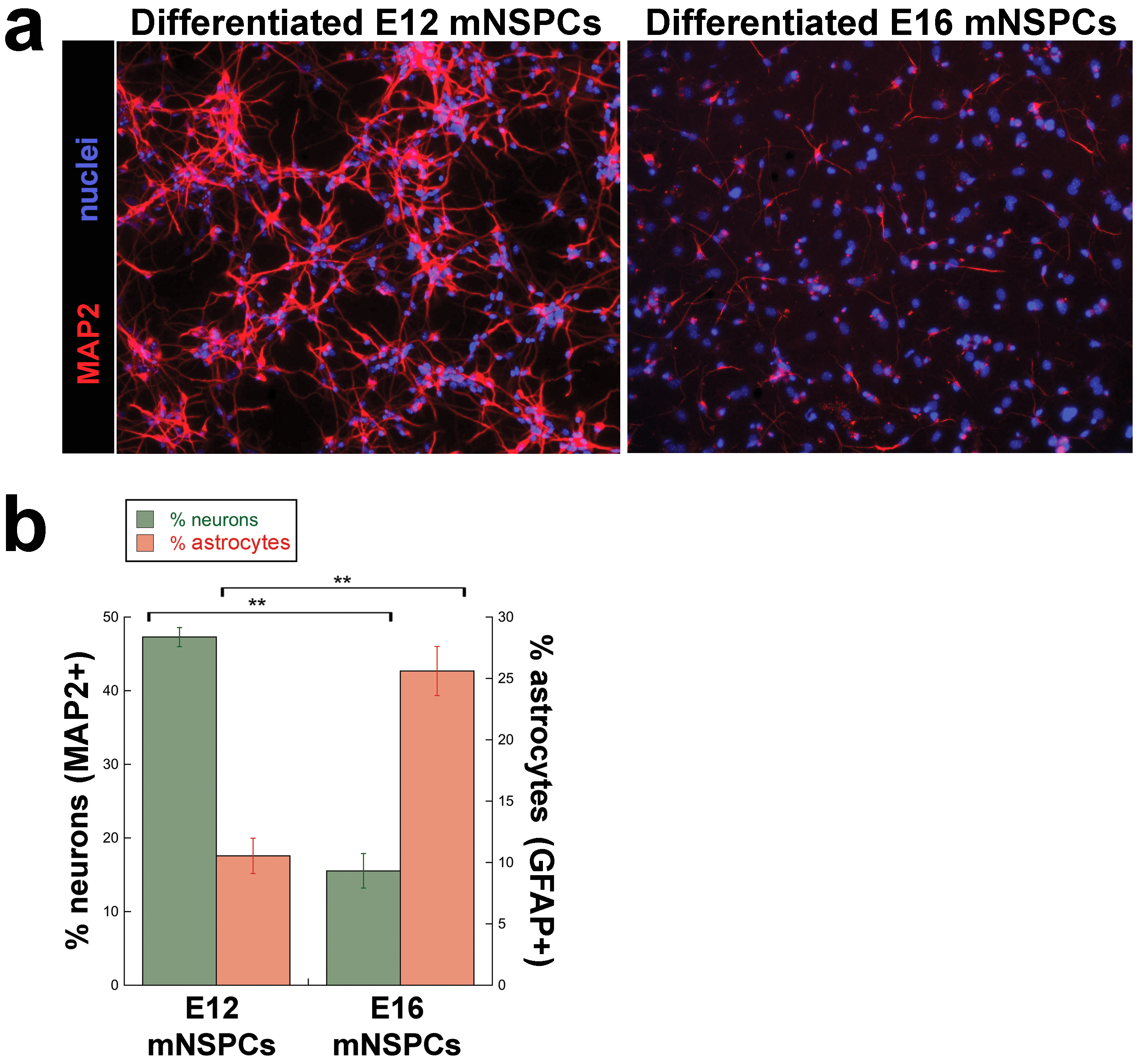

Supplement: Figure S2 — Mouse NSPCs (mNSPCs) from distinct stages of development (E12 and E16) differ in neurogenic capacity. (a) Immunostaining of differentiated mNSPCs from distinct developmental stages with antibodies to neuronal markers reveals that more MAP2-positive neurons are generated from E12 mNSPCs than E16 cells. Similar results were obtained with antibodies for the neuronal markers TuJ1 and doublecortin (data not shown). (b) The percentages of differentiated MAP2-positive neurons and GFAP-positive astrocytes generated from E12 and E16 mNSPCs shows that E12 cells are more neurogenic and E16 cells more gliogenic. Neuron and astrocyte counts were obtained from cells differentiated for 3 days (**p<0.01, n = 250 or more cells from 3 separate experiments). Error bars represent s.e.m. (TIF) [file pone.0025458.s002.tif]

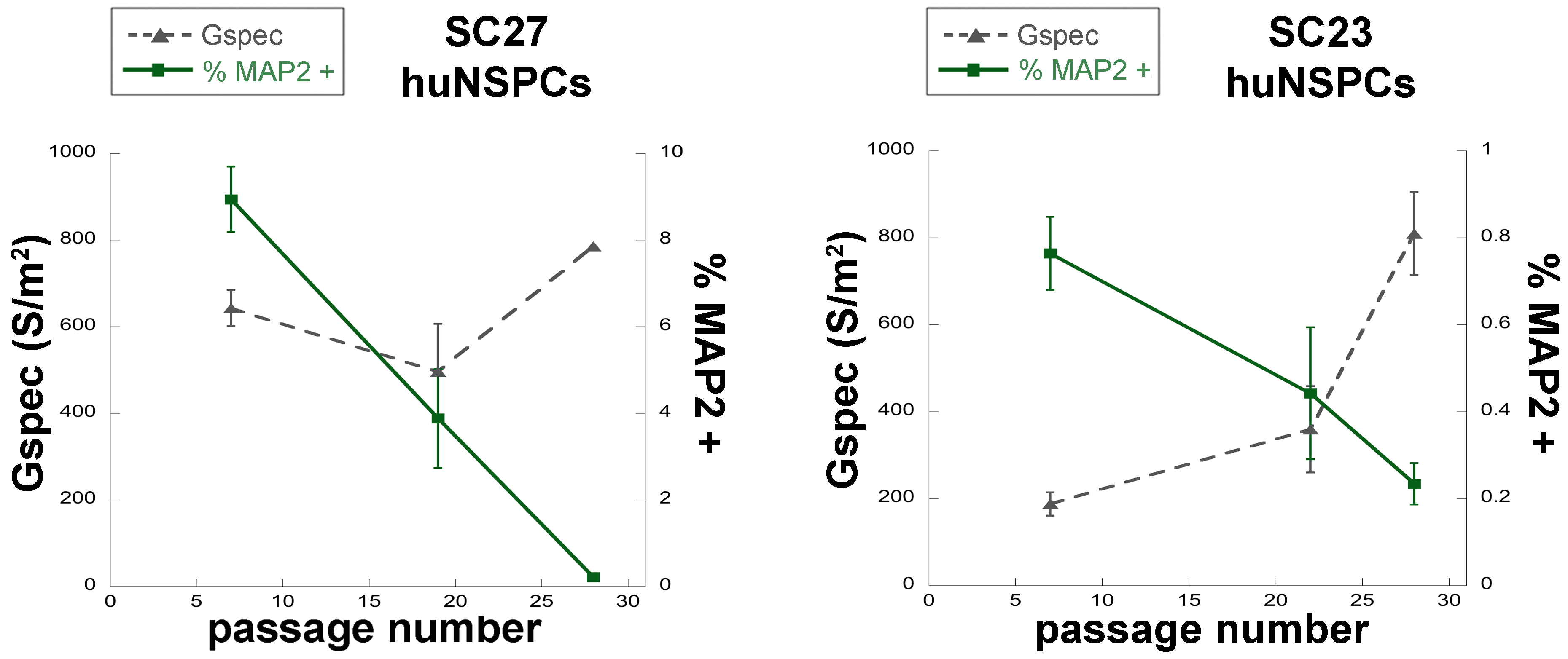

Supplement: Figure S3 — HuNSPC membrane capacitance does not consistently correlate with neurogenic potential. Graphs of specific membrane conductance (Gspec, S = Siemens) and neurogenic capacity of SC27 or SC23 cells (revealed by generation of MAP2-positive neurons) over increasing passage number demonstrate the lack of a clear correlation between these measures. Gspec values of SC27 cells begin high, drop lower, and increase again as the neurogenic potential decreases. SC23 cells display a similar decrease in neurogenic potential over increasing passage number but their Gspec values begin low, increase slightly, and increase again. Error bars represent s.e.m. and n = 3 or more separate experiments with different sets of cells. (TIF) [file pone.0025458.s003.tif]
